# Experimental and agent-based simulation analysis of collective escape in mice: Validation and density-dependent effects of obstacles

**DOI:** 10.1371/journal.pone.0355105

**Published:** 2026-08-04

**Authors:** Duyen Thi Hai Nguyen, Taehyeong Kim, Junyoung Park

**Affiliations:** 1 Department of Mechanical System Engineering, Kumoh National Institute of Technology, Gumi, Gyeongbuk, Korea; 2 Department of Aeronautic, Mechanical and Electrical Convergence Engineering, Kumoh National Institute of Technology, Gumi, Gyeongbuk, Korea; 3 Department of Mechanical Engineering, Kumoh National Institute of Technology, Gumi, Gyeongbuk, Korea; International Institute of Information Technology Bangalore, INDIA

## Abstract

Understanding collective movement under stress provides insight into the mechanisms governing escape dynamics. This study investigated how multiple environmental and group factors jointly affect the escape dynamics of mice under emergency-like conditions induced by electrical stimuli. The tested factors included stimulus intensity (40–55 V), the presence or absence of an obstacle near the exit, the guide walls at different exit angles (15°, 45°, and 75°), and group size (15 vs. 50 mice). Evacuation time, average velocity, and headway were quantified to assess escape efficiency. To validate and extend the experimental findings, an agent-based modeling framework—previously verified in similar studies—was used to reproduce and generalize the tested conditions. Results show that the effects of obstacles and guide walls depend strongly on both group size and stimulus intensity: under high-density and moderate-stimulus conditions, obstacles and guide walls facilitated smoother flow and shorter evacuation times, whereas under low-density or extreme stimuli, they hindered movement. These results demonstrate the density-dependent nature of obstacle effects and confirm that headway serves as a robust mechanistic indicator of flow stability, revealing that sufficient inter-agent spacing is essential to prevent physical interlocking and clogging at the bottleneck. Overall, this study integrates experiment and simulation to establish a validated framework for analyzing collective evacuation dynamics in animals under controlled conditions.

## 1. Introduction

### 1.1. Motivation

Collective movement under emergency conditions has long been a critical topic in safety science. In large gatherings, uncoordinated escape behavior can lead to congestion, clogging, and even fatal stampedes. Recent disasters, such as the 2021 Meron festival in Israel, the 2022 Kanjuruhan stadium incident in Indonesia, and the Itaewon tragedy in South Korea, tragically demonstrate how uncontrolled or high-pressure crowd flow can result in severe casualties [1–3]. These events underscore the urgent need to understand the mechanisms that govern collective escape under high-stress conditions.

Despite extensive research efforts, reproducing and analyzing crowd-crush phenomena remains highly challenging in both empirical and computational studies. In controlled experiments, the primary difficulty lies in replicating high-stress evacuation interactions without compromising participant safety. In numerical simulations, the challenge arises from the multifactorial nature of human movement. It is affected by psychological responses under emergency evacuation, physical contact forces, frictional interactions, and body deformation under compression. Moreover, when individuals traverse bottleneck regions, even minute variations in personal velocity or intent can amplify nonlinearly, leading to abrupt slowdowns of the collective flow.

### 1.2. Experimental and simulation approaches for studying collective escape dynamics

Previous experimental investigations on collective escape behavior have been conducted using both human participants and animal models. Experiments involving humans provide direct observations of evacuation behavior [4–11]. However, conducting such experiments requires rigorous ethical review and strict safety protocols, which significantly limit the number and scale of human studies. As a result, empirical data on human crowd behavior under high-stress evacuation conditions remain scarce, making it difficult to fully capture the complex mechanisms governing collective escape.

To address this limitation, animal experiments have emerged as a valuable alternative, allowing for controlled and repeatable studies under stress-induced conditions. Various species have been employed, including mice [12–23], sheep [24,25], and ants [26,27], each offering distinct advantages and limitations. Among these, mice have become particularly popular due to several favorable characteristics. First, mice are cognitively capable and exhibit behavioral responses to fear or stress that share qualitative similarities with human escape dynamics [28,29]. They also demonstrate self-organized queuing and effects of prior individual training or warning, which have been used to investigate collective mechanisms analogous to those in human evacuations [18,20]. The density–velocity–flow relationships in mice have been reported to show similarities to those in human pedestrian data, supporting their use as an experimental system for exploring evacuation-related dynamics [14]. Second, the cost and logistical demands of housing and training mice are relatively low compared with larger animals such as sheep or cattle. Third, their small body size enables flexible experimental design and easier environmental control. Consequently, mice have been widely used as an experimental system for studying collective motion under emergency conditions.

Various stimuli have been applied to induce escape behavior in rodent experiments, including water immersion [13,18,21], smoke exposure [15,16,22], and electric shocks [12,29–31]. While all methods can effectively elicit escape responses under highly stressful emergency conditions, electric stimulation offers a practical advantage: it enables precise and continuous adjustment of the stimulus intensity, facilitating controlled comparisons across experimental conditions.

In parallel with animal experiments, computational modeling has become an indispensable approach for exploring evacuation phenomena under controlled virtual conditions. While experiments provide direct observations, they are limited in the range of parameters that can be systematically explored. Simulation complements this by enabling controlled variation of conditions and offering insight into underlying mechanisms that are difficult to isolate experimentally. Numerous evacuation models have been developed to study the movement characteristics and emergent phenomena that occur during crowd evacuation. These models can be classified into three main types: cellular automata (CA) models [32–35], macroscopic continuum models [36–40] and social force models (SFM) [41–45]. CA models simulate pedestrian movement at a microscopic level, optimizing evacuation space design and assessing obstacles’ impact. Macroscopic continuum models treat pedestrian flow as a fluid-like system, capturing collective behavior at high densities. SFM effectively models self-organization in crowds, investigating congestion under highly stressful emergency conditions and transitions from free-flow to congestion.

With further advances in computational methods, researchers have increasingly adopted agent-based modeling (ABM) frameworks to represent both physical interactions and behavioral decision-making in evacuation scenarios. Within this broader ABM family, Discrete Element Method (DEM)-based formulations serve as one specific approach for resolving contact forces between individuals and boundaries. Several DEM-based and agent-based approaches have been developed to capture different aspects of crowd dynamics under high-density conditions. For instance, the CrowdDMX framework focuses on subgroup formation and collective behavior within crowds, highlighting the importance of social grouping in evacuation scenarios [46]. In addition, various psychological extensions have been proposed to incorporate behavioral effects relevant to emergency scenarios, including models with multiple psychological components [47], extended 2D movement [48] and applications to guide-wall studies [31,49]. Furthermore, another DEM-based multi-agent model, referred to as the CBS-DE model, has been developed to simulate crowd behavior in various scenarios, including tsunami shelters [50], destination-switching [51], and self-avoidance via an avoidance algorithm [52]. However, each model also presents limitations. The model that incorporates multiple psychological effects struggles with curved corridors and high-density avoidance. CrowdDMX and CBS-DE excel at avoidance routines but pay less attention to other psychological components critical under emergency conditions. The ABM used in this study integrates social-force behavioral rules, DEM-based contact mechanics, and a CrowdDMX-style avoidance algorithm, thereby enhancing both physical realism and behavioral responsiveness during evacuation.

Despite these advances, important gaps remain in existing agent-based simulation approaches. Previous studies have often emphasized global outcomes such as average velocity and evacuation time [53,54], but have rarely explored how local organizational patterns—particularly headway distributions and inter-agent spacing—govern evacuation efficiency [55–57]. Furthermore, while some works have examined avoidance behaviors and architectural features, the systematic influence of simultaneous obstacle placement and guiding-wall installation, as well as their dependence on crowd density, has not been comprehensively addressed [24,58].

Reducing congestion at bottleneck exits has been a major research objective. Placing obstacles near exits is one proposed strategy, but its effectiveness remains controversial. Some studies indicate that appropriately placed obstacles can shorten evacuation time in human experiments [8], reduce local pressure and alleviate congestion between the obstacle and the exit [59], induce proactive avoidance behavior during the early stages of evacuation [10]. In contrast, other studies have observed that the presence of obstacles can impede escape performance, resulting in lower average speeds and longer total evacuation times [60]. It is important to note that most of these experiments were conducted with human participants, where the sense of urgency was simulated through verbal or instructional cues rather than actual highly stressful emergency conditions.

Experiments involving animal models have provided additional insights into this issue. Studies with sheep have demonstrated that flow dynamics are highly sensitive to obstacle placement, and that there may exist an optimal configuration depending on crowd density and environmental geometry [61]. Similarly, in mouse experiments, the influence of obstacles on evacuation efficiency has been found to depend strongly on both the size of the obstacle and its distance from the exit [17].

Overall, although significant progress has been made in both experimental and simulation-based approaches, the mechanisms underlying local organization and obstacle effects during collective escape remain incompletely understood. These unresolved issues motivate the investigation presented in the following section.

### 1.3. Proposed solution and contributions

Despite extensive research on crowd evacuation, no previous study has systematically investigated how multiple environmental factors—such as stimulus intensity, obstacle placement, angle of guide wall (exit angle), and group size—jointly influence collective escape dynamics. Understanding these interactions is crucial, as real emergency situations often involve the simultaneous presence of several influencing factors. However, because of the nonlinear nature of crowd behavior, the combined effects cannot be inferred simply by summing individual contributions. This motivates the need for controlled experiments that capture the co-occurrence of multiple variables during evacuation.

In this study, both experimental and numerical approaches are employed to examine collective escape under combined conditions. The experimental component involves groups of mice subjected to electrical stimulation on the paws to induce escape responses. The choice of animal model and stimulus type has been justified in the preceding section. Experimental observations were used to characterize behavioral trends and to contribute to the estimation of simulation parameters, in combination with previously reported results [12]. In addition, the experimental results serve as a reference for validating the consistency of the simulation outcomes.

On the modeling side, an ABM integrating social-force behavioral rules with DEM-based contact mechanics was adopted and further refined by incorporating an obstacle-avoidance algorithm analogous to wall-avoidance behavior, as well as headway-based metrics to characterize microscopic self-organization. A series of evacuation scenarios were then simulated with variations in exit geometry, stimulus intensity, crowd size, and obstacle configuration. The simulation framework enables exploration of a wider range of conditions, including larger group sizes and diverse obstacle placements, which are difficult to systematically investigate through animal experiments.

The results reveal systematic variations in evacuation speed, total escape time, and local headway distributions, providing new insight into the mechanisms underlying the Faster-Is-Slower (FIS) phenomenon. The simulation outcomes are compared with experimental observations to assess consistency, while also extending the analysis by revealing underlying mechanisms that cannot be directly observed in experiments.

Overall, this work contributes to the field by:

(1) conducting integrated mouse experiments and agent-based simulations to analyze evacuation under combined environmental influences;
(2) introducing a headway-based analytical framework for linking local interaction patterns with global evacuation efficiency; and
(3) providing empirical and computational evidence that can inform obstacle placement and exit design for mitigating congestion during emergency evacuations.

## 2. Materials and method

### 2.1. Ethics statement

All experimental procedures involving live vertebrate animals were reviewed and approved by the Institutional Animal Care and Use Committee (IACUC) of the Ulsan National Institute of Science and Technology (UNIST) under Authorization No. UNISTIACUC-16–21. All animal handling and experimental protocols strictly adhered to the IACUC guidelines for the care and use of laboratory animals to ensure humane treatment throughout the study. Reporting of animal research follows the ARRIVE (Animal Research: Reporting of In Vivo Experiments) guidelines to promote transparency and reproducibility.

**Methods of sacrifice:** No animals were euthanized during experimental procedures. Following completion of the experiments, animals were transferred to and managed by trained personnel at the institutional animal facility in accordance with the approved IACUC protocol (Authorization No. UNISTIACUC-16–21) and institutional guidelines.

Methods of anesthesia and/or analgesia: No anesthesia or analgesia was administered during behavioral testing because the objective of the study was to assess natural escape responses, and these interventions could alter behavioral outcomes.

**Efforts to alleviate suffering:** To minimize animal distress, the electrical stimulus was carefully controlled within a predefined range (40–55 V), and all experiments were conducted for short durations. Mice underwent a habituation period prior to testing, and their condition was continuously monitored throughout the experiments. Between trials, mice were allowed sufficient rest periods and were provided with free access to food and water to minimize fatigue and stress accumulation.

### 2.2 Experiment setup and procedure

Previous studies on pedestrian evacuation have often been constrained by ethical and safety concerns, limiting the ability to reproduce emergency conditions with human participants. To address this, mice were used as surrogate agents for humans, and electrical stimulation was applied as a controlled proxy for emergency stress, compelling the animals to evacuate [21,28]. Although electrical shocks do not mimic real-world emergencies exactly, they offer a quantifiable and repeatable method for inducing urgency in laboratory settings [29]. A total of 50 mice (25 males and 25 females) were obtained from the In Vivo Research Center at the Ulsan National Institute of Science and Technology (UNIST), South Korea. The mice ranged from 9 to 11 months of age, with average body dimensions of 2.5–3.0 cm (width), 7.8–9.8 cm (length), and 2.6–3.0 cm (height), and weights ranging from 25 to 35 grams. For the 15-mouse experimental group, 8 males and 7 females were selected.

**Fig 1:**
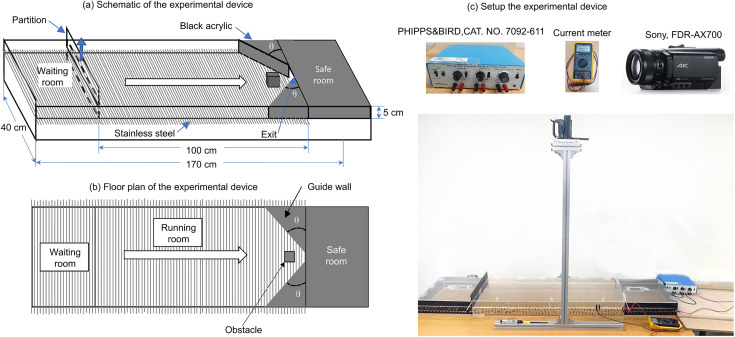
Experimental apparatus and layout. (a) Schematic of experimental device. (b) Floor plan of the experimental device. (c) Photograph of the experimental setup.

The experimental setup consisted of a custom-designed structure comprising three zones: a waiting room, a running room, and a safety room (Fig 1.(a) and (b)). Although the total length of the apparatus is 170 cm, only the running room (40 cm × 100 cm) was used as the computational (analysis) domain for extracting and analyzing evacuation data. A Phipps & Bird Isolated Square Wave Stimulator (Model 7092−61) delivered electrical stimuli to the waiting and running rooms. The narrow exit between the running room and the safety room was a fixed 5 cm wide opening. A guide wall was installed near the exit, and its orientation angle was varied at 15°, 45°, and 75° to assess the effect of exit angle. Additionally, in the trials examining obstacle effects, the obstacle was positioned differently for each exit-angle configuration. For each exit angle, the obstacle was therefore positioned along the corridor centerline at a distance from the nominal exit such that the effective passage width on both sides matched approximately two body widths of the mice. This design ensured that the o bstacle did not over-restrict capacity at low densities, while still perturbing the approach geometry to the exit. Based on this design, the corresponding obstacle positions were approximately 7 cm from the exit for the 15° and 45° exit-angle configurations, and 19 cm for the 75° exit-angle configuration.

All mice underwent a pre-experiment habituation session to reduce novelty-induced responses, following established procedures in prior studies [12]. Two density levels were tested: 15-mouse group (low density); 50-mouse group (high density). For each condition, trials were conducted under combinations of: guide-wall angle (15°, 45°, 75°), presence or absence of the obstacle, and electrical stimulus amplitudes incrementally increased from 40 V to 55 V in 5-V steps [12].

At the beginning of each trial, mice were placed in the waiting room with the partition closed. After verifying the preset conditions (stimulus amplitude, guide-wall angle, obstacle configuration), electrical stimulation was applied. The partition was then opened to initiate the evacuation. A trial ended when all mice had entered the safety room. All experiments were recorded using a digital camcorder. Each experimental condition was repeated three times. The reported values are presented as mean ± standard deviation across the three repeated trials (n = 3). Because of the limited number of replicates, these statistics are intended primarily for qualitative comparison of trends and should not be interpreted as evidence of statistical significance. The same group of mice was used across different experimental conditions. This reuse may introduce potential learning or habituation effects. To minimize such effects, sufficient rest periods were provided between consecutive trials, during which mice had free access to food and water. In addition, the order of experimental conditions was randomized.

**Fig 2:**
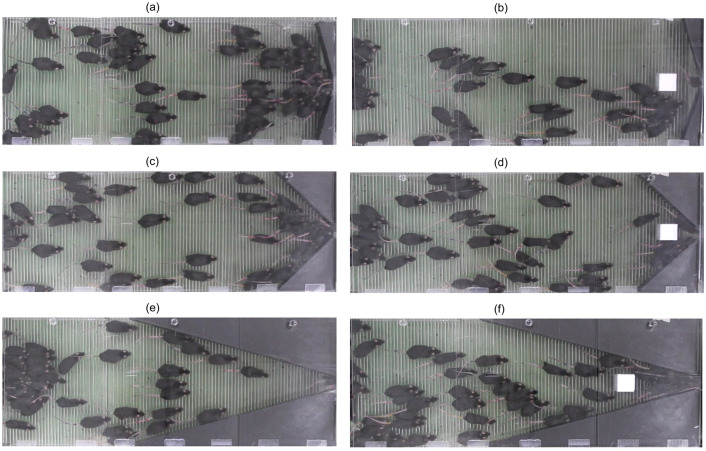
Representative top-view snapshots at 5 s after partition opening (stimulus 40 V). Snapshots show guide wall angles and obstacle presence: (a) 15° no obstacle; (b) 15° with obstacle; (c) 45° no obstacle; (d) 45° with obstacle; (e) 75° no obstacle; (f) 75° with obstacle.

Video recordings were analyzed using MouseTracker, an in-house software tool developed for frame-by-frame tracking. Video files were converted into image sequences, and the position of each mouse was extracted in every frame. Behavioral quantities, including velocity and total evacuation time, were computed using definitions and analysis methods adapted from Oh’s previous work [31]. Representative snapshots taken 5 seconds after initiating evacuation at a 40-V stimulus are shown in Fig 2. (a) 15° no obstacle; (b) 15° with obstacle; (c) 45° no obstacle; (d) 45° with obstacle; (e) 75° no obstacle; (f) 75° with obstacle. Experimental datasets used in this study are publicly available on Zenodo [62].

### 2.3. Simulation

To capture the evacuation dynamics of mice in experiments, we simulated their movement using an ABM, which has been validated in previous studies [31]. In this framework, the Discrete Element Method (DEM) governs the fundamental physical interactions between agents and between agents and walls or obstacles, whereas the Social Force Model (SFM) accounts for behavioral intent and decision-making.

The motion of each agent 𝑖 is governed by Newton’s second law:


$$\begin{array}{c} {\text{m}}_{\text{i}}\frac{{\text{d}}^{\text{2}}{\vec{\text{r}}}_{\text{i}}}{\text{d}{\text{t}}^{\text{2}}}={\vec{\text{F}}}_{\text{i}}^{\text{contact}}+{\vec{\text{F}}}_{\text{i}}^{\text{psy}}, \end{array}$$ (1)

where ${\text{m}}_{\text{i}}\;$is the agent’s mass, ${\vec{\text{r}}}_{\text{i}}$ denotes the position vector of agent i, ${\vec{\text{F}}}_{\text{i}}^{\text{contact}}\;$is the physical contact force arising from interactions, ${\vec{\text{F}}}_{\text{i}}^{\text{psy}}\;$represents the psychological/social force.

**Fig 3:**
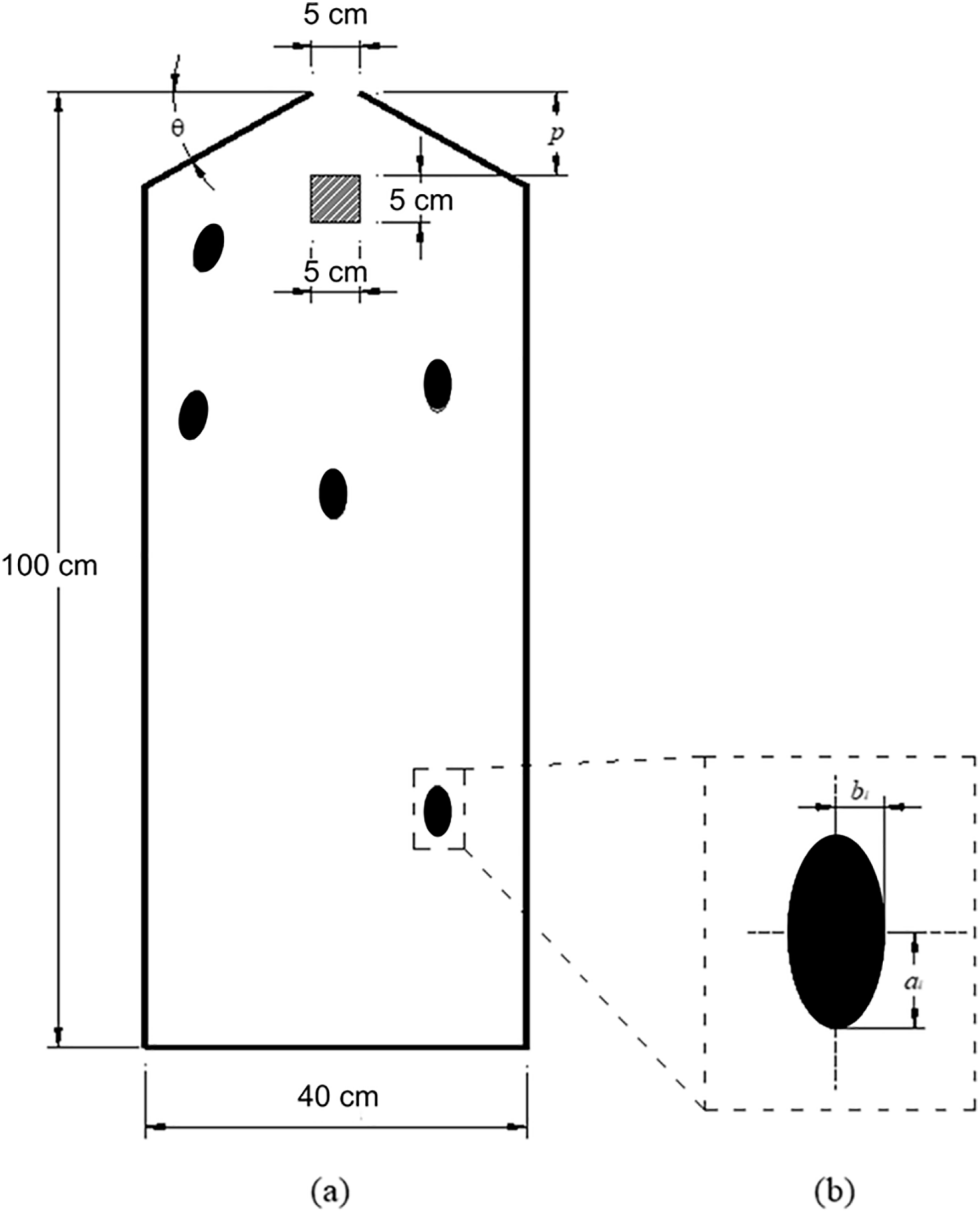
Simulation geometry and agent model. (a) Running-room geometry used in simulations, showing exit position, obstacle, and guide-wall angle definition. (b) Ellipse representation of a mouse used in the model, defined by semi-major and semi-minor axes (a_i, b_i) = (2.5, 1.25) cm.

Contact force (DEM component): Collisions are modeled with a soft-particle spring–dashpot formulation.


$$\begin{array}{c} {\vec{\text{F}}}_{\text{ij}}^{\text{contact}}=\text{k}\delta_{\text{ij}}{\vec{\text{n}}}_{\text{ij}}-\eta \left({\vec{\text{v}}}_{\text{ij }}.{\vec{\text{ n}}}_{\text{ij}}\right){\vec{\text{n}}}_{\text{ij}}, \end{array}$$ (2)

where k is the spring constant, $\delta_{\text{ij}}$ is the overlap distance, η is the damping coefficient, ${\vec{\text{n}}}_{\text{ij}}$ is the unit normal vector, and ${\vec{\text{v}}}_{\text{ij }}$is the relative velocity vector. Simulation parameters are summarized in Table 1. Each agent is represented as an ellipse to reflect body anisotropy, defined by semi-major and semi-minor axes of 2.5 cm and 1.25 cm, respectively (Fig 3(b)). Overlap detection between agents follows Ueda’s algorithm [48]. Specifically, the agent width (2.5 cm) is determined based on experimental observations, where the exit width (5 cm) allows approximately two mice to pass simultaneously. The agent length (L = 5 cm) is chosen to provide a consistent interaction scale in the direction of motion. Under crowded conditions, the effective occupied length of mice is reduced due to body compression and overlap, and the selected value ensures realistic spacing and interaction behavior.

**Table 1:** Simulation parameters.

Parameter	Value
Spring constant (k)	$\text{0}.\text{5}\;\times \;{\text{10}}^{\text{5}}$ N/m
Restitution coefficient	0.1
Friction coefficient	0.1
Time step (Δt)	0.001 s
Exit angle (θ)	${\text{15}}^{\text{0}}$
Number of agent	10, 15, 20, 30, 50, 100, 150, 200
Agent weight	30g
Agent size (semi-axes, ${\text{a}}_{\text{i}};{\text{b}}_{\text{i}}$)	(2.5, 1.25) cm

Psychological force (SFM component): Behavioral tendencies are modeled using the classical social-force formulation.


$$\begin{array}{c} {\vec{\text{F}}}_{\text{i}}^{\text{psy}}=\frac{{\text{m}}_{\text{i}}}{\tau }\left({\vec{\text{v}}}_{\text{i}}^{\text{des}}{-\vec{\text{v}}}_{\text{i}}\right), \end{array}$$ (3)

where ${\vec{\text{v}}}_{\text{i}}^{\text{des}}$ is the desired velocity vector, ${\vec{\text{v}}}_{\text{i}}$ is the current velocity, and τ is the response time.

The desired velocity of each agent consists of both a magnitude and a direction. The direction was determined by incorporating psychological decision-making rules observed in emergency evacuations. In such situations, agents attempt to escape rapidly, often competing for limited exit space and exhibiting reduced collision avoidance, which can lead to congestion and clogging near bottlenecks [8,24,25]. To replicate this behavior, four behavioral conditions were implemented: (i) rushing toward the exit, (ii) overtaking slower agents ahead, (iii) stepping away from the nearest wall, and (iv) avoiding side-by-side alignment with neighboring agents. The desired direction of each agent was thus determined based on these psychological rules, as described by Oh et al. [31]. The magnitude of the desired velocity and its standard deviation were determined based on a combination of experimental observations from the present study and previously reported results by Nguyen et al. [12], which provide a baseline dataset characterizing velocity responses under different stimulus intensities. This approach ensures consistency between the simulation inputs and experimentally observed behavior, while avoiding direct parameter fitting from a single experimental dataset. The effect of different stimulus intensities is incorporated implicitly through velocity adaptation, rather than introducing a separate explicit stimulus parameter.

In this study, the original model was extended to include an obstacle placed in front of a narrow exit. The obstacle was positioned at a distance p from the exit, and this parameter was systematically varied to analyze the effect of obstacle placement for each simulated agent size. The tested distances were p = 0L, 1L, 2L, 3L, 4L, 5L, and 6L, where L represents the agent length (L = 5 cm). A value of p = 0L corresponds to the no-obstacle condition, while larger values indicate obstacle placement at increasing multiples of the agent’s body length.

To accurately depict real scenarios, an obstacle collision-avoidance algorithm was implemented similarly to the method used for wall avoidance. The simulation domain of the running chamber was configured with the specified exit angle and obstacle placement, as shown in Fig 3.(a). The simulation model and source code used for validation and extended analyses are openly available at Zenodo [62].

**Fig 4:**
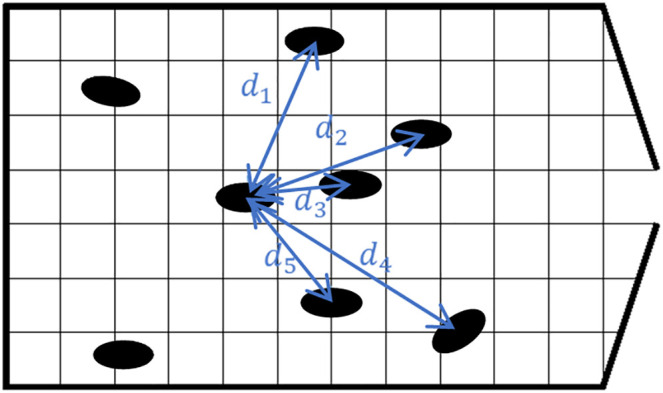
Definition of headway. Headway for agent i is defined as the minimum distance from agent i to the nearest agent ahead in the running direction.

Headway analysis – beyond global measures (velocity and evacuation time), we compute the headway, defined as the minimum distance from agent i to the nearest agent in front (Fig 4). This variable captures local spacing patterns and explains counterintuitive evacuation outcomes such as the Faster-is-Slower effect.

## 3. Results

### 3.1. Effect of obstacle and mouse number

**Fig 5:**
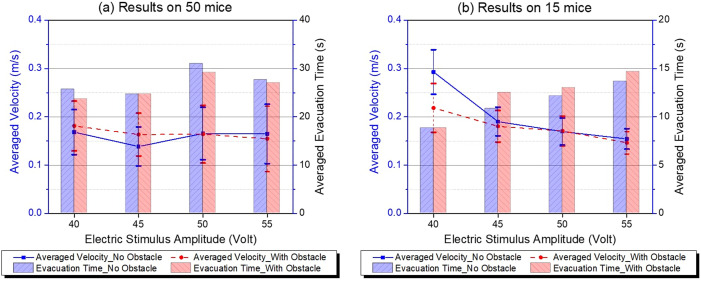
Experimental comparison of obstacle effect with 15° guide wall. (a) Averaged velocity and evacuation time for high-density group (50 mice) and (b) low-density group (15 mice) across stimulus amplitudes (40–55 V, step 5 V). Error bars represent mean ± standard deviation across three repeated trials (n = 3).

Fig 5 illustrates the effect of placing an obstacle in front of a narrow exit on evacuation dynamics under different electric stimulus amplitudes in both high-density (50 mice) and low-density (15 mice) groups.

In the high-density condition (Fig 5.(a)), the presence of an obstacle tended to reduce the average evacuation time across most tested stimulus levels. These observations are consistent with previous studies reporting density-dependent effects and flow improvement under similar conditions [24,53,57]. Although changes in average velocity were relatively small, the decrease in evacuation time suggests that the obstacle functioned as a flow-regulating element, reducing clogging near the exit. At 50V and 55V, evacuation time was shorter with the obstacle despite similar velocities, indicating that improved spatial organization, rather than speed, contributed to more efficient outflow. At 45V, the average velocity in the presence of an obstacle was higher than without it, while the evacuation time were nearly identical. This implies that under moderate stimulus intensity, the obstacle may facilitate more movement, resulting in increased velocities without an apparent effect on overall evacuation time.

In contrast, the low-density group (Fig 5.(b)) exhibited a different pattern. The presence of an obstacle generally increased evacuation time and reduced velocity as the stimulus amplitude increased. The only exception occurred at 40V, where evacuation time was slightly lower with the obstacle. This result may suggest that, in low-density scenarios, the obstacle acted as a hindrance, possibly due to insufficient crowd pressure to require flow regulation. The similarity in evacuation time at higher stimulus levels, despite lower velocity with the obstacle, further supports the idea that the obstacle reduced evacuation efficiency in low-density groups.

The Faster-Is-Slower (FIS) effect, which refers to the counterintuitive phenomenon where increasing individuals’ desired velocity leads to slower evacuation, is partially observed in the high-density condition shown in Fig 5.(a). As the electrical stimulus amplitude increases from 45V to 50V, both the average velocity and evacuation time increase simultaneously. This goes against the usual pattern where higher velocity leads to shorter evacuation time. It suggests that when individuals try to move faster due to stronger stimuli, it can cause more crowding near the exit and slow down the overall evacuation. A similar trend is observed when the amplitude increases from 50V to 55V, where both the average velocity and evacuation time decrease. The fact that evacuation becomes faster even as individuals move slightly slower on average indicates a possible reduction in clogging at extremely high stimulus levels, potentially due to better flow coordination or reduced local interactions caused by dispersion. In contrast, no such effect is observed in the low-density group (Fig 5.(b)). The average velocity tends to decrease while the evacuation time increases as the stimulus amplitude rises. This opposite trend between velocity and evacuation time reflects the typical relationship, where slower movement leads to longer evacuation. This result may suggest that the FIS effect is strongly density-dependent, requiring a sufficient level of crowd interaction and physical interference to emerge.

### 3.2. Effect of exit angle (angle of guide wall)

**Fig 6:**
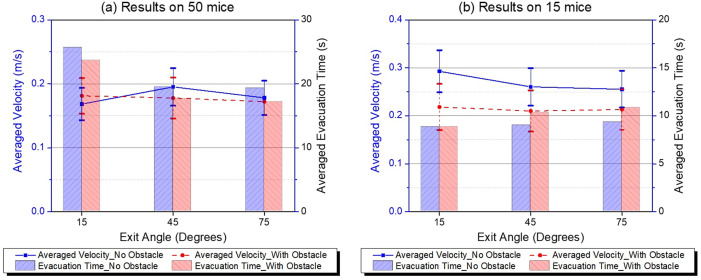
Effect of exit angle on experimental outcomes at 40V. (a) High-density group (50 mice). (b) Low-density group (15 mice). Plots show mean evacuation time and mean velocity with and without the obstacle. Error bars represent mean ± standard deviation across three repeated trials (n = 3).

Fig 6 presents the experimental results for both high-density (50 mice, Fig 6.(a)) and low-density (15 mice, Fig 6.(b)) conditions when varying the exit angle under a constant stimulus amplitude of 40V, with and without the presence of an obstacle.

In the high-density group (Fig 6.(a)), increasing the exit angle from 15° to 75° resulted in a noticeable decrease in average evacuation time, indicating improved evacuation efficiency. This effect is more pronounced when the obstacle is present. Across all exit angles, the average evacuation time is consistently shorter in the presence of the obstacle than without it, demonstrating that the combination of an obstacle and angled guide walls can effectively regulate flow and reduce congestion near the exit. At an exit angle of 15°, the obstacle also led to higher average velocity and shorter evacuation time, as expected. However, at 45° and 75°, the FIS effect is observed: although average velocity is higher without the obstacle, the evacuation time is longer compared to the condition with the obstacle. This result may suggest that in high-density scenarios, increased individual velocity does not always lead to faster evacuation, likely due to congestion effects near the exit.

In contrast, the low-density group (Fig 6.(b)) shows a different tendency. As the exit angle increases, average velocity decreases and evacuation time increases, regardless of the presence of an obstacle. These trends are consistent with the typical inverse relationship between velocity and evacuation time and show no evidence of the FIS effect. Furthermore, the presence of the obstacle appears to hinder evacuation under all exit angles: velocities are lower and evacuation times are longer when the obstacle is present. This again emphasizes the density-dependent nature of obstacle effectiveness. Under low-density evacuation conditions, obstacles and guide walls may impede rather than improve the evacuation process.

### 3.3. Simulation results and comparison with experiments

Simulation based on pedestrian behavior models is widely used to study evacuation dynamics, especially when real-world experiments are limited by ethical or practical constraints. To ensure the reliability of such models, it is essential to validate them against experimental observations. In this context, the following section presents the simulation results obtained using an ABM, which have been refined and compared with the experimental results discussed earlier. This comparison aims to evaluate the model’s ability to reproduce the dynamic behaviors observed in the experiments and to reinforce the validity of the experimental findings.

**Fig 7:**
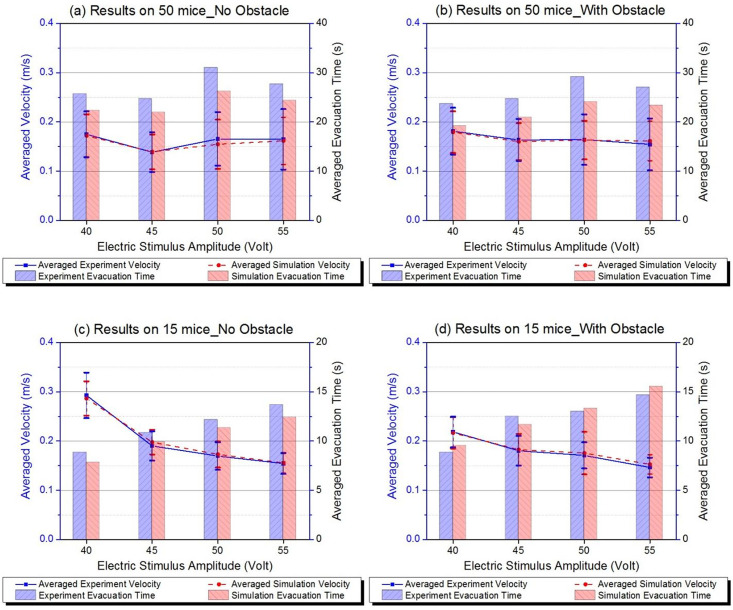
Comparison between experiments and simulations across stimulus amplitudes. (a) Results on 50 mice-No Obstacle. (b) Results on 50 mice-Obstacle. (c) Results on 15 mice-No Obstacle. (d) Results on 15 mice- Obstacle. Error bars represent mean ± standard deviation across three repeated trials (n = 3).

Fig 7 illustrates the similarity between experimental and simulation results in analyzing the effect of stimulus amplitude on the average velocity of mice in both groups. The error bars indicate the standard deviation of velocity, and the differences between experimental and simulation results are minimal. The simulation closely reproduces both the average velocity and its variation under different stimulus conditions, with and without obstacles in front of the narrow exit. The figure also presents the average evacuation time for 50 and 15 mice. While the simulation does not match the experimental values exactly, it successfully captures the same trend in evacuation time observed in the experiments for both group sizes. These results suggest that, although the simulation does not replicate the exact numerical outcomes, it reliably reproduces the experimental trends and can be used to validate and support experimental findings.

**Fig 8:**
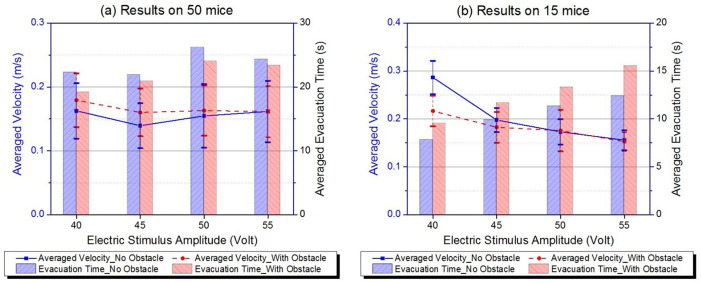
Simulation results showing obstacle effect. (a) High-density case (50 agents). (b) Low-density case (15 agents). Error bars represent mean ± standard deviation across three repeated trials (n = 3).

Fig 8 shows the simulation results comparing evacuation with and without an obstacle at the exit, under high-density (50 mice, Fig 8.(a)) and low-density (15 mice, Fig 8.(b)) conditions. In the high-density case, the average evacuation time is shorter when the obstacle is present across all stimulus amplitudes. In contrast, in the low-density case, evacuation time increases and average velocity decreases in the presence of the obstacle. These simulation outcomes correspond closely with the experimental results in Fig 5. In both simulations and experiments, the obstacle contributes to reduced evacuation time under high-density conditions, while it has the opposite effect under low-density conditions. The consistency between the two approaches supports the applicability of the simulation model in reproducing density-dependent evacuation behavior.

### 3.4 Simulation expansion

a. Effect of obstacle in evacuation when the number of agents changes.

**Fig 9:**
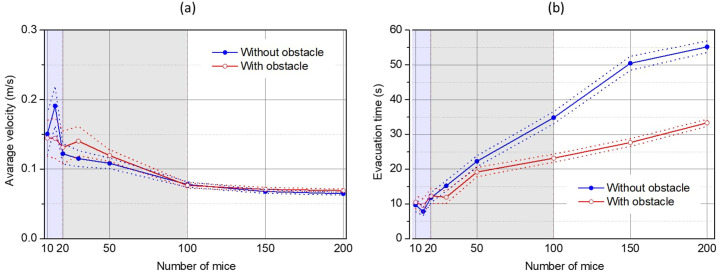
Simulation expansion: effect of the obstacle across different group sizes, with and without an obstacle. (a) Averaged velocity. (b) Total evacuation time. The dashed lines indicate the standard deviation of the data.

To explore the effect of obstacles on evacuation efficiency, we performed simulations with agent populations ranging from 10 to 200, comparing scenarios with and without an obstacle positioned in front of the narrow exit. Fig 9 (a) illustrates how average velocity varied with population size under scenarios with and without an obstacle. As expected, increasing the number of agents led to a general decrease in average velocity in both cases. This trend primarily reflects the constraint imposed by the fixed simulation domain: as population size increases, the available space per individual diminishes, forcing agents to slow down regardless of their desired speed. Beyond this overall trend, three distinct density regimes emerge when comparing the two scenarios. At low densities (≤30 agents), the difference of average velocity between the obstacle and no-obstacle cases is irregular and lacks a systematic trend. With few interactions and minimal congestion, the effect of the obstacle depends largely on the instantaneous positions of individuals near the exit, leading to fluctuating outcomes.

In the intermediate density range (30–100 agents), the obstacle begins to show a stabilizing influence. Here, the obstacle scenario consistently maintains slightly higher average velocities than the no-obstacle case. This improvement likely arises from the obstacle’s ability to disrupt small dense clusters that form directly in front of the exit. Compared to the no-obstacle case, these local congestions are noticeably reduced. As a result, the overall flow remains smoother under moderate crowding. At very high densities (≥100 agents), the average velocity in both scenarios converges to similarly low values because space limitations and frequent interactions dominate the entire domain. In this regime, the obstacle does not appear to substantially alter the average velocity, as overall movement is constrained throughout the space. However, this should not be interpreted as a loss of effectiveness in improving evacuation outcomes. On the contrary, as shown in Fig 9.(b), the obstacle’s localized regulation of flow near the exit becomes increasingly crucial at high densities, substantially reducing evacuation time despite the low average velocity.

Fig 9 (b) shows the evacuation time for increasing population sizes in both scenarios. At low densities (≤30 agents), the presence of an obstacle slightly increased evacuation time. In this regime, where congestion is virtually absent, the obstacle introduces a minor detour and localized delays near the exit without offering compensatory benefits in regulating flow. As density increased beyond this threshold, a distinct shift in evacuation dynamics emerged. For intermediate to high densities (30–100 agents), the obstacle consistently reduces evacuation time, with the improvement becoming more pronounced as density rises. The underlying mechanism lies in its ability to redistribute approaching flows and prevent excessive clustering at the narrow exit. By mitigating bottleneck pressure, the obstacle transforms the evacuation process from an unstable stop-and-go pattern into a more continuous and efficient flow. At very high densities (≥100 agents), the advantage of the obstacle peaks, with evacuation times reduced by over 40% compared to the no-obstacle scenario. This shows that carefully placing obstacles can help reduce severe congestion.

**Fig 10:**
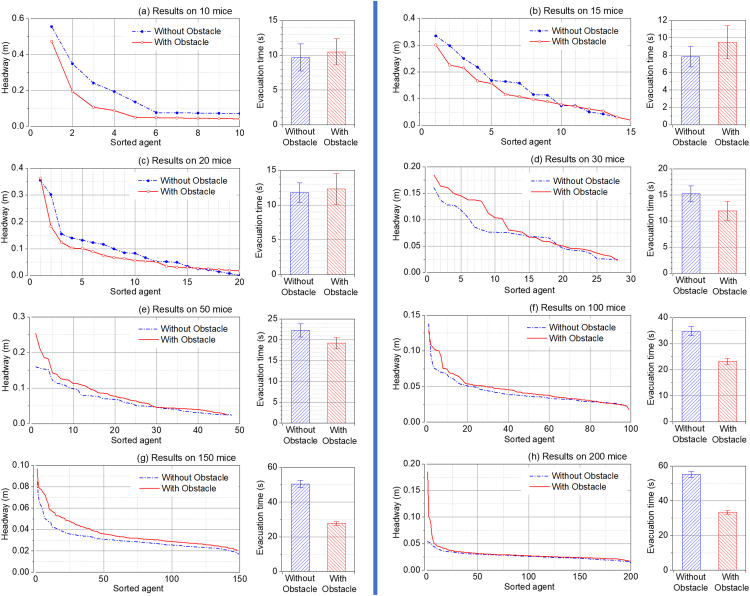
Headway analysis across densities. (a–c) Headway distributions for low-density cases (<30 agents). (d–h) Headway distributions for intermediate and high densities (30–200 agents). Each panel compares obstacle and no-obstacle conditions. Agents are sorted by decreasing headway; thus, the x-axis reflects only their rank order.

Figs 10 present the distributions of average headway for different group sizes. As expected, headway decreases monotonically with increasing group size, reflecting the natural reduction in inter-agent spacing as more animals occupy the confined space. Besides, the presence of an obstacle near the exit induces a distinct density-dependent influence on spacing behavior.

For low-density conditions (< 30 agents; Fig 10.(a)–(c)), the obstacle slightly reduces headway compared to the no-obstacle case, accompanied by longer evacuation times. This result may suggest that, under low-density conditions, the obstacle disrupts the flow by introducing an additional barrier, which hinders the coordination and movement of agent. The effect is most pronounced when the crowd size is minimal, where spatial freedom is high and any obstruction directly interferes with optimal movement. In contrast, at medium to high densities (≥ 30 agents; Fig 10. (d)–(h)), the obstacle increases the average headway and shortens evacuation time. At higher densities, the obstacle mitigates congestion at the exit by preventing the formation of interlocked clusters dominated by contact forces. By redirecting flow and promoting more uniform spacing, the obstacle enables a smoother, more organized evacuation, thereby increasing the effective speed of movement. This effect becomes increasingly pronounced as the number of agents rises, with scenarios involving 100–200 individuals showing a tendency toward shorter evacuation times, even though differences in headway gradually diminish at extreme densities.

Overall, these observations demonstrate that average headway provides a meaningful indicator of evacuation efficiency. Larger headway corresponds to smoother flow, minimized physical overlap and frictional interlocking, and a more stable movement sequence, all of which contribute to shorter evacuation times. Conversely, reduced headway is associated with increased interactions and local congestion that slow down the overall evacuation process. Importantly, the magnitude and sign of obstacle-induced changes in headway depend strongly on the density: obstacles tend to hinder movement when the group is sparse but may improve organization and alleviate clogging when the crowd is moderately to highly packed.

b. Effect of changing obstacle position on total evacuation time

**Fig 11:**
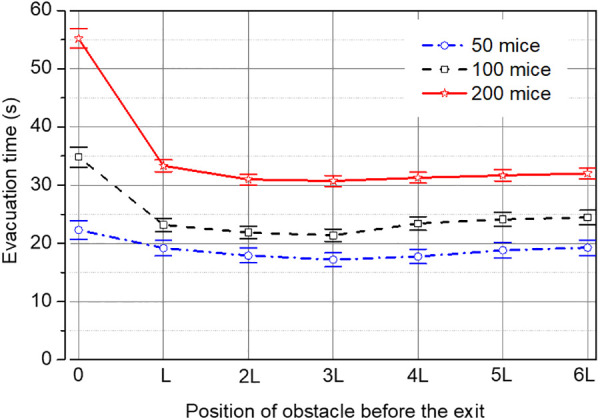
Effect of obstacle position on evacuation time for different group sizes.

Placing an obstacle in front of a narrow exit has proven effective in enhancing evacuation at high densities. To optimize this strategy, it is essential to investigate how the obstacle’s position relative to the exit influences performance. Fig 11 demonstrates that all three population sizes exhibit a similar non-linear response to obstacle placement: evacuation times initially decrease as the obstacle is moved away from the exit (from 0L to 3L), reach a minimum at an intermediate distance (p = 3L), and then increase again as the obstacle is placed farther away (≥4L).

The optimal placement is found at p = 3L from the exit, where evacuation times are minimized across all densities. This distance corresponds to 3 times the body length of the agent. From a granular mechanics perspective, this specific spatial scale is critical [63]. A distance shorter than one body length (e.g., 1L) causes the obstacle to merge with the bottleneck, restricting the effective width. Conversely, a distance that is too large allows the re-formation of stable arches (force chains) between the obstacle and the exit. Therefore, at p = 3L, the obstacle acts as an effective ‘arch-breaker’ that disrupts the formation of dense clusters before they solidify at the exit, while still maintaining sufficient guiding pressure to channel individuals smoothly toward the opening.

A second key finding is that evacuation time does not scale linearly with the number of evacuees. While increasing the number of agents from 50 to 200 quadruples group size, evacuation time rises only by a factor of approximately 1.2–1.5. This sublinear scaling suggests emergent collective effects within the group: as density increases, interactions among individuals promote self-organization, leading to more efficient use of space and sustained flow even under crowded conditions.

Together, these results highlight two critical insights: (i) obstacle placement is a powerful but highly sensitive intervention, capable of transforming evacuation dynamics if optimally configured, and (ii) collective organization can offset some of the detrimental impacts of high density, demonstrating that group-level behavior plays a pivotal role in evacuation efficiency.

## 4. Discussion

This study combines controlled laboratory experiments with with agent-based simulations to examine how electrical stimulus intensity, crowd density, and the combined use of obstacles and guide walls evacuation dynamics. The results suggest a clear density dependent effect. When the density is high, these structural interventions tend to increase the flow. When the density is low, the same interventions may hinder movement. This section discuss possible behavioral mechanisms behind these patterns. It also consider how well the experiments and simulations correspond and relate the observations to previous studies on collective movement.

*Density-dependent effects of stimulus and inter-individual interactions:* Experimental results show that evacuation behavior is strongly influenced by density-related factors. In high-density conditions (50 mice), a localized Faster-Is-Slower (FIS) effect emerged, characterized by instances where evacuation time and average velocity changed in the same direction. This result may suggest that higher stimulus levels may intensify competitive interactions and produce transient congestion near the exit. In contrast, low-density groups (15 mice) exhibited no FIS effect, either globally or in any stimulus interval. These findings suggest that the FIS effect does not come from stimulus intensity by itself. Instead, it emerges when crowding causes small disturbances to grow into delays that affect the entire group.

*Contrasting effects of obstacles and guiding walls at different densities:* The presence of an obstacle near the exit produced opposite outcomes depending on density. Under high-density conditions, the obstacle reduced evacuation time by reducing crowd build up at the bottleneck and by lowering the pressure concentrated at the center of the exit. In low-density scenarios, however, the same obstacle acted primarily as a barrier, forcing unnecessary detours and increasing evacuation time. Similar density-dependent trends were observed for the guiding wall. Larger exit angles improved evacuation under high-density conditions, especially when combined with an obstacle, but at low density these interventions slowed movement. These contrasting outcomes underscore the need for context specific evaluation. An intervention that enhances evacuation in dense conditions may hinder performance when there is plenty of space.

*Simulation-based validation and extended analysis*: agent-based simulations were conducted to evaluate whether the model could reproduce the evacuation observed in the experiments. Across both low- and high-density conditions, the simulation results captured the same qualitative trends reported in the mouse experiments, including the density-dependent influence of obstacles and the effects of electrical stimulus amplitude. Although the numerical values of velocity and evacuation time were not identical to the experimental measurements, the consistency of the trends supports the validity of the agent-based simulations framework for representing physically driven interactions in this system. Beyond validation, the simulations were used to explore conditions that were not experimentally tested. Analysis of headway revealed a clear correspondence between larger inter-agent spacing and more efficient evacuation, mirroring the experimental observations. This result may suggest that headway may function as a useful dynamic indicator of flow stability within this model. However, beyond serving as an indicator, headway plays a critical mechanistic role in the transition from fluid-like flow to clogging.

Specifically, when the headway decreases below a critical threshold (approaching the sum of agent radii), the interaction shifts from psychological avoidance to physical contact. In the context of our DEM formulation (Eq. 2), this vanishing headway triggers immediate particle overlap ($\delta_{\text{ij}}$), generating significant repulsive forces (${\vec{\text{F}}}_{\text{ij}}^{\text{contact}}$) and frictional interlocking. Consequently, the kinetic energy of agents is dissipated through damping and collision rather than contributing to forward motion, facilitating the formation of stable force chains (arching) at the bottleneck. Therefore, the obstacle’s effectiveness at high densities can be physically interpreted as a mechanism that forcefully resets the headway distribution, preventing these contact forces from dominating the flow.

The extended simulations also allowed examination of obstacle effects over a broader range of densities and obstacle positions. As the number of simulated agents increased, the obstacle exhibited a transition from being ineffective at low densities to substantially improving evacuation performance at moderate and high densities. Furthermore, sweeping the obstacle position identified an intermediate distance equal to three times the agent length (3L), that consistently minimized evacuation time across densities, suggesting that spatial placement is an important design parameter. These simulation outcomes do not establish the detailed mechanisms underlying the observed patterns but provide a controlled means to explore how density and obstacle configuration interact within the model.

*Limitations and future works:* Together with the experimental findings, these simulation results provide a consistent picture of density-dependent evacuation behavior and offer a basis for future studies aimed at quantifying the evacuation processes. Although the combined experiments and simulations give a clear picture of how density affects evacuation in mice, some limitations remain. The study’s generality is constrained by the restricted geometric conditions tested and the model’s focus on physical rather than cognitive aspects of behavior. Nevertheless, the agreement between simulations and experiments suggests that this approach is useful for examining interaction-driven evacuation dynamics. Future work may explore different obstacle geometries, multi-exit designs, and broader density ranges, as well as extend the validated modeling framework to human-oriented simulations for hypothesis testing. Overall, this study provides a coherent set of observations showing that the effects of obstacles and guide walls depend strongly on density, and that headway is a useful indicator of flow stability within this system.

## 5. Conclusion

This study combined controlled mouse experiments with agent-based simulations to examine how electrical stimulus intensity, group density, and exit-side architectural features shape evacuation dynamics. The results show that evacuation efficiency strongly depends on density. Under high-density conditions, both obstacles and guide walls improved performance by preventing clogging and reducing evacuation time, and localized Faster-Is-Slower effects were observed. At low densities, however, the same structures created unnecessary detours and prolonged evacuation. Agreement between experiments and simulations indicates that the agent-based simulations framework captures observed behaviors and allows investigation of situations beyond experimental reach. Headway emerged as a robust indicator of flow stability and a key mechanistic determinant. The study demonstrates that maintaining sufficient inter-agent spacing effectively minimizes the physical contact forces and frictional interlocking, thereby preventing the formation of force chains that lead to clogging. Extended simulations further revealed an optimal obstacle placement, three times the agent length from the exit, that maximized these regulatory effects. Overall, these findings advance the understanding of density-dependent evacuation behavior, demonstrate how small geometric interventions can meaningfully improve performance, and provide a practical basis for optimizing architectural layouts and guiding future research on collective escape dynamics under high-stress conditions.
